# Optimization Studies on Compression Coated Floating-Pulsatile Drug Delivery of Bisoprolol

**DOI:** 10.1155/2013/801769

**Published:** 2013-11-10

**Authors:** Swati C. Jagdale, Nilesh A. Bari, Bhanudas S. Kuchekar, Aniruddha R. Chabukswar

**Affiliations:** Department of Pharmaceutics, MAEER's Maharashtra Institute of Pharmacy, MIT Campus, Survey No. 124, Kothrud, Pune, Maharashtra 411 038, India

## Abstract

The purpose of the present work was to design and optimize compression coated floating pulsatile drug delivery systems of bisoprolol. Floating pulsatile concept was applied to increase the gastric residence of the dosage form having lag phase followed by a burst release. The prepared system consisted of two parts: a core tablet containing the active ingredient and an erodible outer shell with gas generating agent. The rapid release core tablet (RRCT) was prepared by using superdisintegrants with active ingredient. Press coating of optimized RRCT was done by polymer. A 3^2^ full factorial design was used for optimization. The amount of Polyox WSR205 and Polyox WSR N12K was selected as independent variables. Lag period, drug release, and swelling index were selected as dependent variables. Floating pulsatile release formulation (FPRT) F13 at level 0 (55 mg) for Polyox WSR205 and level +1 (65 mg) for Polyox WSR N12K showed lag time of 4 h with >90% drug release. The data were statistically analyzed using ANOVA, and *P* < 0.05 was statistically significant. Release kinetics of the optimized formulation best fitted the zero order model. *In vivo* study confirms burst effect at 4 h in indicating the optimization of the dosage form.

## 1. Introduction

Due to poor drug efficacy, the incidence of side effects, and frequency of administration to conventional drug preparations, many traditional drug dosage forms are undergoing replacement by second-generation, modified drug release dosage forms. During the early 1990s, second-generation modified release drug preparations achieved continuous and constant rate drug delivery, in which constant or sustained drug output minimize drug concentration “peak and valley” levels in the blood, promoting drug efficacy and reducing adverse effects [1, 2].

Recent studies also reveal that the body's biological rhythm may affect normal physiological function, including gastrointestinal motility, gastric acid secretion, gastrointestinal blood flow, renal blood flow, hepatic blood flow, urinary pH, cardiac output, drug-protein binding, and liver enzymatic activity, and biological functions such as heart rate, blood pressure, body temperature, blood plasma concentration, intraocular pressure, stroke volume, and platelet aggregation [3]. Most organ functions vary with the time of the day, particularly when there are rhythmic and temporal patterns in the manifestation of a given disease state. The symptoms of many diseases, such as bronchial asthma, myocardial infarction, angina pectoris, hypertension, and rheumatic disease have followed the body's biological rhythm [4–6]. Day night variation in asthmatic dyspnea and variations in the incidence of myocardial infarction occur throughout the early morning hours.

A chronodelivery system, based on biological rhythms, is a state-of-the-art technology for drug delivery; chronomodulated DDSs not only increase safety and efficacy levels, but also improve overall drug performance [7, 8]. The time-controlled function of third generation DDSs currently under development is finding application in new and improved disease therapeutics. Biological rhythms may be applied to pharmacotherapy by adopting a dosage form that synchronizes drug concentrations to rhythms in disease activity [9, 10]. 

Chronotherapeutics refers to a treatment method in which *in vivo* drug availability is timed to match rhythms of disease, in order to optimize therapeutic outcomes and minimize side effects [11]. Pulsatile drug delivery systems can be classified into site-specific systems in which the drug is released at the desired site within the intestinal tract (e.g., the colon) or time-controlled devices in which the drug is released after a well-defined time period [12, 13]. 

The human body has many built-in rhythms known as biological clocks. Ultradian cycles are shorter than a day. Circadian cycles last about 24 hours. Circadian phase dependent patterns have been well documented in conditions such as asthma, arthritis, epilepsy, migraine, allergic rhinitis, cardiovascular disease (myocardial infarction, angina, and stroke) and peptic ulcer disease, with particular times where symptoms are more prominent and/or exacerbated. Treating these diseases with immediate release dosage forms may be impractical if the symptoms of the disease are pronounced during the night or early morning [14–17].

Bisoprolol fumarate is a cardio selective *β*1-adrenergic blocking agent used for secondary prevention of myocardial infarction (MI), heart failure, angina pectoris, and mild-to-moderate hypertension with half-life 12 h [18]. Polyox are water soluble resins and highly water soluble polymers. Upon exposure to water or gastric juice, they hydrate and swell rapidly to form hydrogels with properties suited for controlled drug delivery. The objective of the present work was to design and optimize compression coated floating-pulsatile system for bisoprolol fumarate using design of experimental study and to find out the best possible formulation [19–22]. 

## 2. Materials and Methods

### 2.1. Materials

Bisoprolol fumarate was a gift from Dr. Reddys Lab. Hyderabad, HPMC K4M, HPMC K100M, and Polyethylene Oxide; that is, Polyox WSR205 and Polyox WSR N12K were gifts from Colorcon Asia Pvt. Ltd. (Goa, India). Croscarmellose sodium and microcrystalline cellulose superdisintegrant were gifts from Vapi Care Pharma. 

### 2.2. Formulation of Rapid Release Tablets by Direct Compression

The inner core tablets were prepared by using direct compression method. As shown in Table 1 powder mixtures of bisoprolol fumarate, microcrystalline cellulose (MCC, Avicel PH-102), croscarmellose sodium (Ac-Di-Sol), and lactose ingredients were dry blended for 20 min, followed by addition of magnesium stearate. Concentration of superdisintegrant croscarmellose sodium varied from 5 to 15% in C1 to C4. The mixtures were then further blended for 10 min. 75 mg of resultant powder blend in minipress 8 station tablet compression machine using 6 mm round concave punch and die to obtain the core tablet.

### 2.3. Preparation of the Floating Pulsatile Release Tablet (FPRT) Trial Batches Individual Polymer

Floating pulsatile release tablets were prepared by press coated method using Polyox WSR205, Polyox WSR N12K polymers, sodium bicarbonate and citric acid as a gas generating agent in batch P1 to P6 as shown in Table 2. Concentration of gas generating agent was varied between 25% (50 mg) of sodium bicarbonate, and 10% (20 mg) citric acid. After finalizing the optimum concentration of gas generating agents, concentration of individual polymer was determined and used in designing the experiment of factorial design. Half of barrier layer material, that is, 50%, was weighed and transferred into an 8.5 mm die; then the core tablet was placed at the center. The remaining half of the barrier layer material was added into the die and compressed [23–28].

### 2.4. Formulation of the Floating-Pulsatile Release Tablet (FPRT)

#### 2.4.1. Experimental Design

A full factorial 3^2^ design was used for optimization procedure. It is suitable for investigating the quadratic response surfaces and for constructing a second-order polynomial model, thus enabling optimization of the time-lagged coating process. Mathematical modeling, evaluation of the ability to fit to the model, and response surface modeling were performed with employing Design-Expert. A 3^2^ randomized reduced factorial design was used in this study and 2 factors were evaluated, each at 3 levels; experimental trials were performed at all 9 possible combinations prepared according to the formula as given in Table 3. The percentage of Polyox WSR205 (*X1*) and Polyox WSR N12K (*X2*) were selected as independent variables. Lag period at 4 h, drug released, and swelling index were selected as dependent variables. The batches thus prepared by factorial design are evaluated and the effect of individual variable is studied according to the response surface methodology [29–35]:

(1) Y=b0+P1X1+P2X2+P12X1X2+P11X12+P22X22,

where *Y* is the dependent variable, *b*_0_ is the arithmetic mean response of the 9 runs, and bi (*P*_1_, *P*_2_, *P*_12_, *P*_11_, and *P*_22_) is the estimated coefficient for the corresponding factor *X*i (*X*_1_, *X*_2_ *, X*_1_ *X*_2_, *X*_12_, and *X*_22_), which represents the average result of changing 1 factor at a time from its low to high value. The interaction term (*X*_1_ *X*_2_) shows how the response changes when 2 factors are simultaneously changed. The polynomial terms (*X*_1_^2^ and *X*_2_^2^) are included to investigate nonlinearity. 

The concentrations of the variables used in the formulation of the tablets of bisoprolol fumarate were decided on the basis of trial batches and their evaluation. The coded levels and the exact concentration of the variables used in different formulations are shown in Table 4.

#### 2.4.2. Preparation of Final Batches

The final batches of the tablets were prepared according to the factorial design. The various batches were prepared according to the concentrations as shown in Table 5.

### 2.5. Formulation of Batches Containing Polyox WSR 205 and Polyox WSR N12K as Variables according to the 3^2^ Full Factorial Design (F10–F18)

The concentration of sodium bicarbonate was kept constant at the optimum level. The optimum level was decided on the basis of the results of the evaluation of trial batches of individual polymer. In this factorial design, concentration of Polyox WSR205 and the concentration of Polyox WSR N12K were varied keeping the values of other ingredients constant. The minimum and maximum levels of the variables were decided on the basis of the predicated individual batches. The concentration of both polymers was finalized in the range of 15 to 30%, so as to study the combined effect of Polyox WSR205 and Polyox WSR N12K on the lag period, release pattern, and swelling index. Tablet batches contain Polyox WSR205 and Polyox N12K as the variables (F10–F18) according to the factorial design showed in Table 5. The effect of the variables on the response was also studied by using the response surface methodology and statistical study by analysis of variance (ANOVA) which was studied by using the Design Expert software (Version 8.0.6). The mathematical modeling and mathematical relationships generated using multiple linear regressions for the studied response variables are expressed in the form of equations.

### 2.6. Manufacturing of Tablets

Respective release retarding polymer Polyox WSR205, Polyox WSR N12K, gas generating agent sodium bicarbonate, and citric acid were weighed and passed through sieve number 20 separately. Powder mixing was carried out using polythene bag for 15 min. Mixing was continued for another 10 min, and rapid release core tablet is prepared according to the formula given in Table 1. Half of barrier layer material was weighed and transferred into an 8.5 mm die then the core tablet was placed at the center. The remaining half of the barrier layer materiel was added into the die and compressed. The hardness of the tablets was adjusted at 7–9 kg/cm^−2^.

### 2.7. Evaluation of Floating-Pulsatile Release Tablet (FPRT)

#### 2.7.1. Physical Evaluation

Tablets were evaluated by various parameters like thickness, hardness, weight, and friability test, % drug content, swelling index, FT-IR study, and DSC.

*Thickness*. Thickness of all tablets was measured using a vernier caliper. 

*Hardness Test*. Monsanto hardness tester was used for the determination of hardness of tablets. The tablet was placed in contact between the plungers and handle was pressed. The force of fractured was recorded. 

*Weight Variation*. The weight of all tablets was taken on electronic balance and the weight variation was calculated. 

*Friability Test*. For each formulation the friability of tablets was determined using the Roche friabilator. In this test tablets were subject to the combined effect of shock abrasion by utilizing a plastic chamber which revolves at a speed of 25 rpm, dropping the tablets to a distance of 6 inches in each revolution. The tablets were then dusted and reweighed. Percent friability (%*F*) was calculated as follows:

(2) $$\begin{array}{c} \%F=\frac{\text{loss}\;\;\text{in}\;\;\text{weight}}{\text{initial}\;\;\text{weight}\;}\times 100. \end{array}$$

#### 2.7.2. Determination of % Drug Content

The tablets were crushed in the mortar and the powder equivalent to 20 mg of drug was dissolved in distilled water. The stock solutions were filtered through a membrane filter (0.45 mm). The solutions were then diluted suitably in distilled water. The drug content was analyzed at 222 nm by UV spectrophotometer (Varian Cary 100). Each sample was analyzed in triplicate.

#### 2.7.3. Drug-Excipient Interactions

The physicochemical compatibilities of the drug and the used excipients were tested by FTIR. FTIR spectra were obtained by using an FTIR spectrometer (Varian, 640IR). Only best formulations F13 were taken into consideration for FTIR study. The drug bisoprolol fumarate and best formulations were previously ground and mixed thoroughly with potassium bromide, an infrared transparent matrix, at 1 : 10 (Sample : KBr) ratio, respectively. The KBr discs were prepared by compressing the powders at a pressure of 5 tons for 5 min in a hydraulic press. Scans were obtained at a resolution of 4 cm^−1^, from 4,000 to 600 cm^−1^.

#### 2.7.4. Differential Scanning Calorimetry (DSC)

Differential scanning calorimetry (DSC) was used to characterize the thermal properties and possibility of any interaction between the excipients and with the drug in physical mixtures. The DSC thermograms were recorded using differential scanning calorimeter (DSC 823e, Mettler Toledo, Switzerland). Approximately 2–5 mg of each sample was heated in a pierced aluminum pan up to 300°C at a heating rate of 10°C/min under a stream of nitrogen at flow rate of 50 mL/min. Thermal data analyses were then done of the DSC thermograms.

#### 2.7.5. Swelling Index Determination

Tablets were weighed individually (designated as *W*_1_) and placed separately in glass beaker containing 200 mL of 0.1 N HCl and incubated at 37°C ± 1°C. At regular 1-h time intervals until 24 h, the tablets were removed from beaker, and the excess surface liquid was removed carefully using the paper. The swollen tablets were then reweighed (*W*_2_) and swelling index (SI) was calculated using the following formula:

(3) $$\begin{array}{c} \text{SI}=\frac{W_{2}-W_{1}}{W_{1}}\times 100. \end{array}$$

### 2.8. *In Vitro* Buoyancy Determination

Floating behavior of the tablet was determined by using USP dissolution apparatus-II in 900 mL of 0.1 N HCl which is maintained at 37 ± 0.5°C, rotated at 50 rpm. The floating lag time as well as total floating time is observed. 

### 2.9. *In Vitro* Drug Release

The release rate was determined for three tablets of each batch using dissolution testing apparatus II (paddle method). The dissolution test was performed using 900 mL of 0.1 N HCl, at 37 ± 0.5°C and 75 rpm. A sample (5 mL) of the solution was withdrawn from the dissolution apparatus till 6 hours at sampling period of 5, 10, 15, 30, 45, and 60 min and then sampling was followed by every twenty minutes till 360 min. Samples were replaced with fresh dissolution medium. The samples were filtered through a 0.45-*μ* membrane filter and diluted to a suitable concentration with 0.1 N HCl. Absorbance of these solutions was measured at 222 nm using Varian cary-100 double beam UV spectroscopy. 

### 2.10. Lag Time

Lag time was considered as the time when the tablet burst and core tablet is out of press coating. This is considered as predetermined off-release period.

### 2.11. *In Vivo* Studies

The *in vivo* X-ray studies of FPRTs were performed on three healthy human volunteers using Simence 300 mA X-ray generating unit. Volunteers aged 25–30 years and weighing 55–60 Kg were selected for these studies. The written consent of the human volunteers was taken before participation and the studies were carried under the supervision of an expert radiologist and physician. Gastric radiography was done at 15 min and 2 and 4 h [36–38].

The formulations F13 were prepared for *in vivo* studies using barium sulphate as radio opaque material. Volunteers were asked to swallow the tablet with sufficient water before the meal under the supervision of physician.

For *in vivo* tests, the tablets with the following composition were compressed.

Formulation F13:

 For core tablet, Barium Sulphate: 20 mg; croscarmellose sodium: 12 mg; magnesium sterate: 3 mg, lactose: 32 mg, microcrystalline cellulose: 08 mg;
 Gas generating agent, sodium bicarbonate: 50 mg; citric acid: 20 mg;
 Erodible outer shell, Polyox WSR205(55 mg), Polyox WSR N12K (65 mg).

### 2.12. Stability Testing of the Best Formulation

A short-term stability study on optimized FPRT was carried out by storing the tablets at 30°C (±2°C) and 65% RH (±5%) and 40°C (±2°C) and 75% RH (±5%) over a 3 months period according to ICH guidelines. At the end of three months' time interval, the tablets were examined for any physical characteristics, drug content, *in vitro* drug release (lag time), floating lag time, and floating duration.

## 3. Results and Discussion

### 3.1. Evaluation of Rapid Release Tablet (RRT)

**Figure 1:**
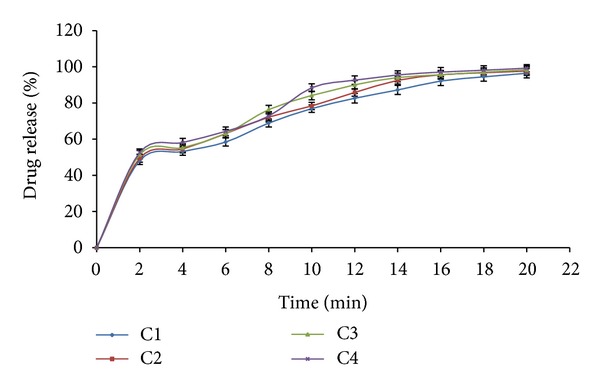
*In vitro* drug release profile of core tablet.

The rapid increase in disintegration of bisoprolol fumarate with croscarmellose sodium in concentration (5–15%) may be attributed to rapid swelling of tablet. It was observed that disintegration time of tablet decrease with increased in concentration of croscarmellose sodium. Formulation C4 (15% croscarmellose sodium) showed lowest disintegration time (66 sec) with high drug release, that is, 99.35% (Figure 1). For development of pulsatile delivery disintegration time must be short to obtain burst effect. The hardness was observed in range of (2.4–2.7 ± 0.18 Kg/cm^2^), whereas friability was less than 1% which indicated that tablet had good mechanical resistance. Drug content was found to be high (>98.14%) and uniform in all tablet formulations. C4 were taken as core tablet for pulsatile release tablet and it was taken for further studies.

### 3.2. Evaluation of Floating Pulsatile Release Tablet (FPRT) Trial Batches Individual Polymers

As concentration of gas generating agent varied, release of drug from formulation was changed. Increase in concentration of sodium bicarbonate affects the release pattern and hardness of the formulation. Concentration of gas generating agent was varied between 25% (50 mg) sodium bicarbonate and 10% (20 mg) citric acid to achieve optimum floating without affecting the release pattern of drug from formulation and to obtain proper lag period. Optimum gas generating agent concentration was 23% (45 mg) for sodium bicarbonate and 8% (15 mg) citric acid. Individual polymer batches (P2 and P5) containing Polyox WSR205 and Polyox WSR N12K in concentration of 70–65% show burst effect after 4 hours after that constant drug release over the period of 6 hours.

**Figure 2:**
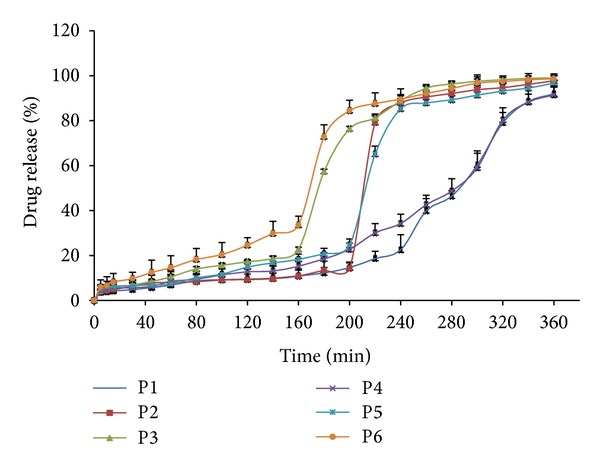
*In vitro* release profiles of batch P1–P6.

From Figure 2 it was observed that compression-coated Polyox WSR205 (P2) and Polyox WSR N12K (P5) when used alone as polymer with croscarmellose sodium (C4) has shown optimal lag period with burst at 3.40 ± 0.2 h and 4.0 ± 0.1 h and drug release 98.97% and 99.16%, respectively. During the dissolution kinetics, the coating layer gradually starts to erode up to a limiting thickness of the coat. After this stage, a rupture of the shell was observed under the pressure which has applied by the swelling of the core tablet due to presence of superdisintegrant. This pressure was high due to high swelling property of croscarmellose sodium, resulted in burst effect after 4 h along with complete and rapid drug release. Formulation P2 and P5 were considered as final batch to study effect of polymers on optimization.

In batches P1 and P4 the amount of coating polymer was too high to achieve high lag time with minimum drug release. P3 and P6 amount of coating polymer was too less which could not maintain integrity of tablet for long and time resulting in complete drug release within short period of time. The drug release clearly depended on the kind and amount of hydrophilic polymer as that which was applied on the core. 

### 3.3. Evaluation of Individual polymers Floating-Pulsatile Release Tablet

Floating-pulsatile release tablet of individual polymer showed (Table 6) tablet weight (260 ± 0.18–275 ± 0.22 mg), thickness (3.50 ± 0.07–3.60 ± 0.04 mm), hardness (7.3 ± 0.16–7.8 ± 0.18 Kg/cm^2^), drug content (>97.48), and buoyancy lag time in range 90–110 sec. 

### 3.4. *In Vitro* Buoyancy Determination

**Figure 3:**
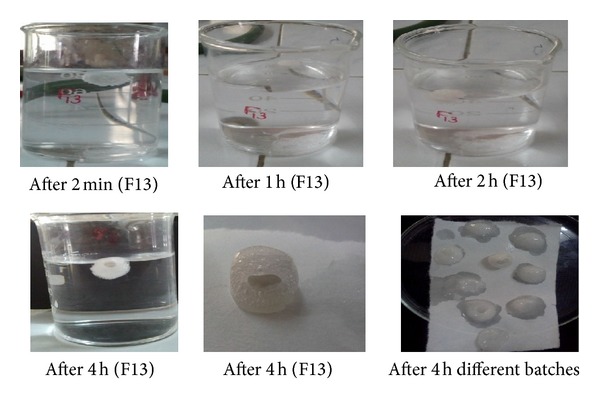
*In vitro* floating behavior of a representative tablet of Polyox WSR.

Floating behavior of tablet depends on added polymers in buoyant layer. Buoyancy lag time was less than 3 min for all batches. Floating time was more than 9 h for all batches.  Figure 3 indicates *in vitro* floating behavior of a representative tablet of Polyox.

### 3.5. Evaluation Combination Polymers of Floating-Pulsatile Release Tablet

Floating-pulsatile release tablet of F10–18 showed tablet weight (291.55 ± 1.34 to 298.78 ± 2.15), thickness (2.44 ± 0.08 to 2.68 ± 0.10 mm), hardness (7.4 ± 0.08 to 8.2 ± 0.34 Kg/cm^2^), drug content (>97.19%), and buoyancy lag time in range 102–119 sec. FPRT of optimized batch F13 showed maximum drug release 99.89 ± 2.01 and less buoyancy lag time 102 sec.

### 3.6. *In Vitro* Drug Release of FPRT according to the 3^2^ Full Factorial Design (F10–F18)

**Figure 4:**
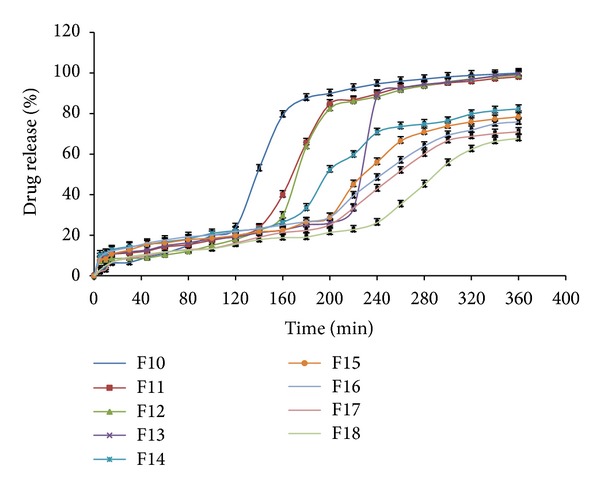
*In vitro* drug release profiles of FPRT of batches F10–F18.

From Figure 4 it was observed that 70 mg Polyox WSR205 (level 0) and 50 mg Polyox WSR N12 K (level +1) in F13 batch have shown lag time of 4.20 hrs, followed by sigmoidal release pattern giving 100% drug release at 6th hour. As the concentration of the Polyox changes from F10 to F18 the lag time and drug release also changes at the 6th hour.

### 3.7. Lag Period

Lag period plays an important role in determining no or less amount drug releases of the tablets at specific period. Ischemic heart diseases, such as angina pectoris and myocardial infarction, are manifested more frequently at peak during the night or early in the morning. Blood pressure which arises notably just before waking up is usually responsible for these attacks. Half-life of bisoprolol is 9 to 12 hr. However for such cases, conventional drug delivery systems are inappropriate for the delivery of Bisoprolol, as they cannot be administered just before the symptoms are worsened, because during this time the patients are asleep. To follow this principle it was necessary to design the dosage form so that it can be given at the bed time giving drug release in the morning. Using current release technology, it was possible to get rapid and transient release of a certain amount of drugs within a short time period immediately after a predetermined off-release period, that is, lag time. Bisoprolol has pH dependent solubility showing better drug bioavailability in stomach, as compared with lower parts of GIT. Overall, these considerations led to the development of oral pulsatile release dosage forms possessing gastric retention capabilities. In designing floating-pulsatile system for bisoprolol with a four-to-five-hour delay in release after oral administration was considered as ideal. The dose administered at bedtime will give drug release in the early morning hours, when the patient is most at risk.

In the formulations from F10 to F18 lag period was in the range of 2–6 ± 0.2 h. In this set of formulation optimum lag period (4.2 ± 0.2) and drug release was observed in the formulation F13. Both polymers (Polyox WSR205 and Polyox WSR N12K) have shown significant effect on lag period. As polymer concentration increase (level 1 to level 1) lag period was also get increased.

The effect of the variables on the lag period in formulations F10–F18 are shown in

(4) Lag  Period=+3.67+1.13A+0.17B+0.1AB−0.40A2+0.30B2

All the polynomial equations were found to be statistically significant (*P* < 0.01), as determined using ANOVA, as per the provision of Design Expert software. 

**Figure 5:**
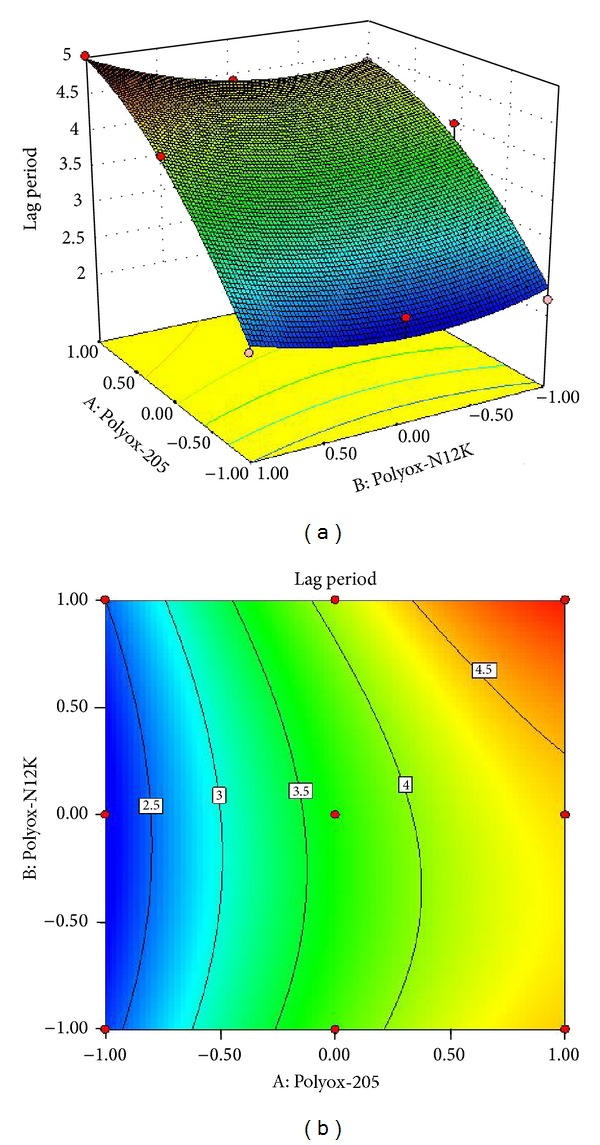
(a) Response surface plot showing the influence on lag period and (b) contour plot.

The combined effect of concentration of Polyox WSR205 and Polyox WSR N12K on lag period is shown in Figure 5. From response surface plot and contour plot in Figure 5, it was observed that both polymers have significant effect on lag period. As concentration of Polyox WSR205 and Polyox WSR N12K increases (level +1, level +1) lag period is increased (>5 h) and uniformed. At maximum level of polymers lag period was increased but did inhibit drug release.

### 3.8. Drug Release

The effect of the variables on the drug release in formulations F10–F18 are shown in

(5) Cumulative  %  Drug  Release   =+72.30−25.86A−10.23B  −3.94AB−6.88A2−0.72B2,

where *A* and *B* represent the variables used in the formulations. 

All the polynomial equations were found to be statistically significant (*P* < 0.01), as determined using ANOVA, as per the provision of Design Expert software. The polynomial equations comprise the coefficients for intercept, first-order main effects, interaction terms, and higher order effects. The sign and magnitude of the main effects signify the relative influence of each factor on the response.

**Figure 6:**
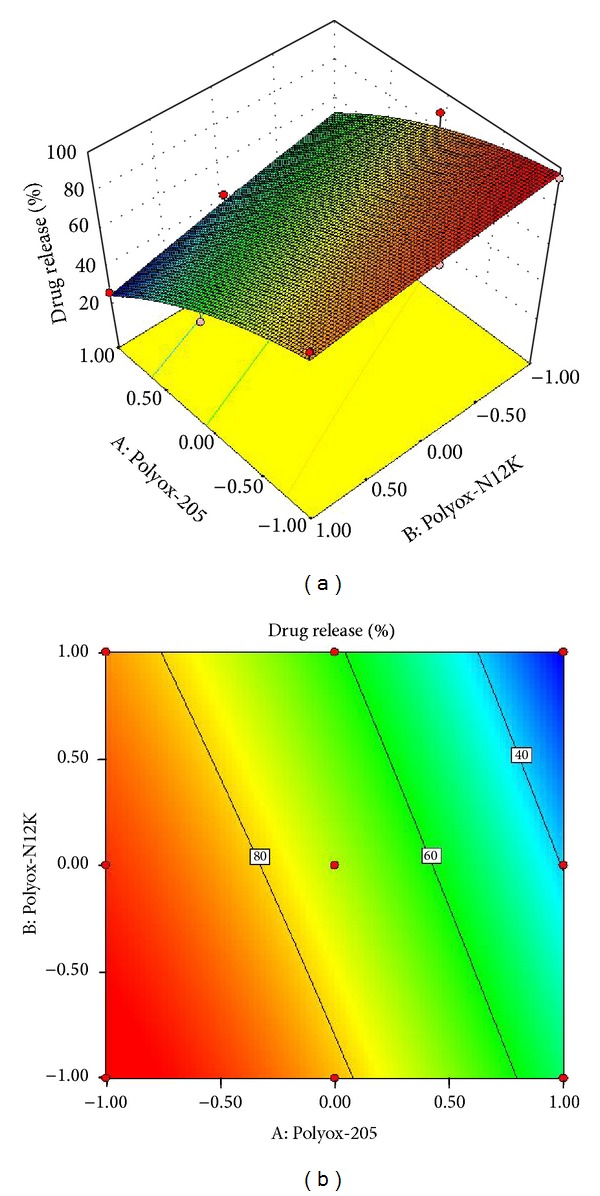
(a) Response surface plot showing the influence on drug release and (b) contour plot.

The combined effect of concentration of Polyox WSR205 and Polyox WSR N12K on drug release was shown in Figure 6. As concentration of Polyox WSR205 and Polyox WSR N12K decreases (level −1, level −1) drug release is higher (>90%) and uniform.

### 3.9. Swelling Index

The swelling index plays an important role in determining the retention ability of the tablets in the stomach. In formulations F10–F18 swelling index was in the range of 113.61 ± 3.13 to 153.13 ± 4.1. In these formulations maximum swelling index (153.13 ± 4.1) has been shown by F16 whereas minimum swelling index (113.61 ± 3.13) by F10. 

The effect of the variables on the swelling index in formulations F10–F18 are shown in

(6) Swelling  index=+137.99+11.91A+3.33B−8.49AB−4.88A2−0.88B2.

All the polynomial equations were found to be statistically significant (*P* < 0.01), as determined using ANOVA, as per the provision of Design Expert software. 

**Figure 7:**
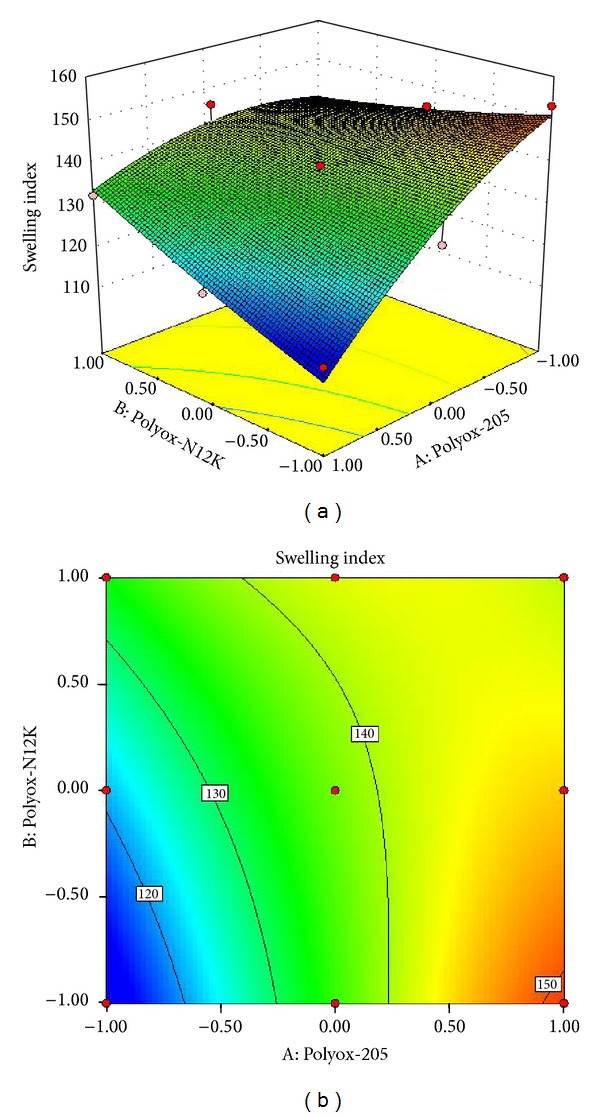
(a) Response surface plot showing the influence on swelling index and (b) contour plot.

The combined effect of concentration of Polyox WSR205 and Polyox WSR N12K on swelling index was shown in Figure 7.

From Figure 7 response surface plot and contour plot, it was observed that increase in the concentration of Polyox WSR205 and Polyox WSR N12K up to intermediate concentration (level 0) there is increase in swelling index.

### 3.10. FTIR Study

**Figure 8:**
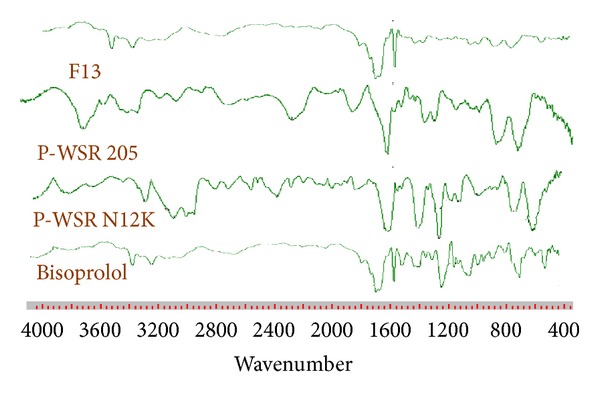
IR spectroscopic study of polymers, drug, and formulations.

There were no interaction between the drug and polymers as showed in Figure 8. IR spectrum of Bisoprolol fumarate is characterized by the absorption of C=O group at 1652 cm^−1^. Polyox WSR-205 has shown major peak at 2959, 1942, and 1482. Polyox WSR N12 K has shown major peak at 3649, 2854, and 1304. Major peaks of polymer are retained in F13 spectra showing no chemical interaction between drug and polymer. In formulation due to cross linking of polymers few bands disappeared and merged. Whereas formulation F13 spectra shown 1652 peak indicating of pure drug and no change in structure of drug.

### 3.11. Differential Scanning Calorimetry

**Figure 9:**
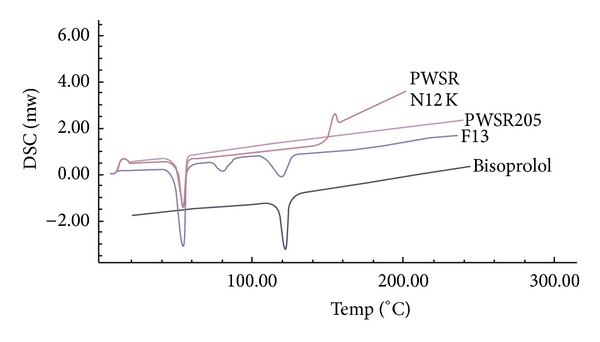
DSC curves of bisoprolol fumarate polymers and formulation F13.

For Bisoprolol fumarate and formulation F13 DSC thermographs is as shown in Figure 9. Thermographs obtained by DSC studies, revealed that the melting point of pure drug is 110°C and for formulations 110°C–112°C. There is no drastic difference in the melting point of the drug as pure and that in the formulations. It can be concluded from this that the drug is in the same pure state even in the formulation. This indicate that there is no chemical interaction between drug and polymer.

### 3.12. * In Vivo* Studies

**Figure 10:**
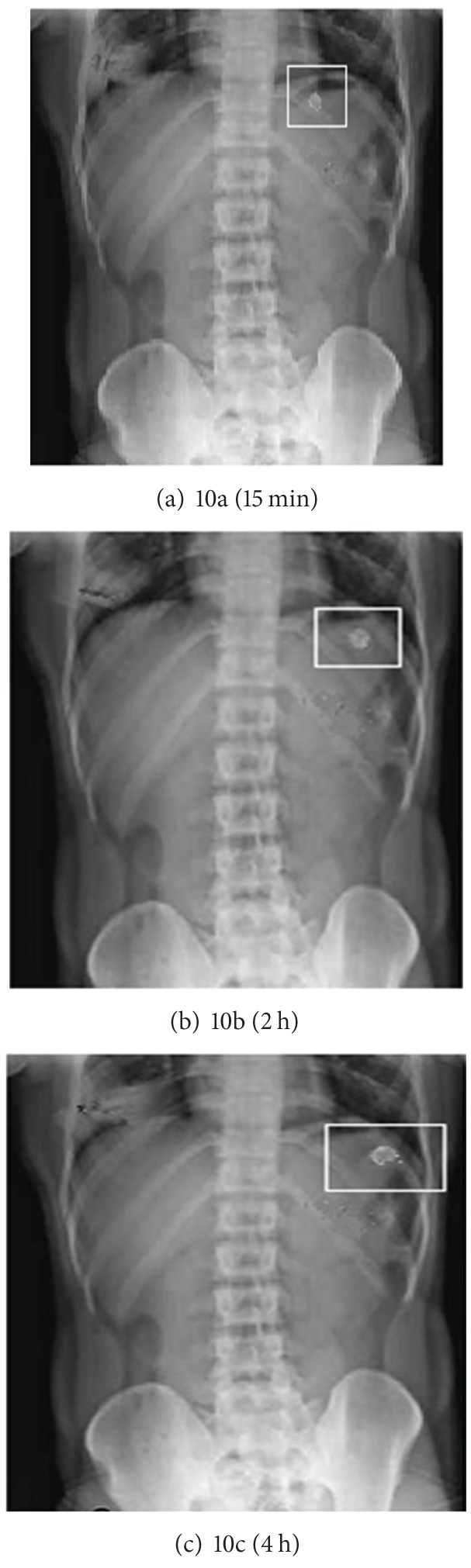
X-ray of formulation F13 at different time intervals.

X-ray taken at 15 min after administration of tablet (F13) is shown in Figure 10(a). Tablet can be seen in the stomach. At 2 h changed in position of tablet; this showed that tablet did not adhere to gastric mucosa in Figure 10(b). Lag time would be completed soon and within short time interval burst effect obtained at 4 h as showed in Figure 10(c). 

### 3.13. Stability Testing of the Best Formulation

F13 optimized batch was selected for the stability studies out of the total formulation batches. For condition 30°C (±2°C) and 65% RH (±5%) and 40°C (±2°C) and 75% RH (±5%) was observed floating time (7.0–7.4 ± 0.8 h) and assay (>97.35%) from initial to 3 months. From stability data it can be concluded that there were no changes in any parameter tested in formulation. From stability data it can be concluded that there were no changes in any parameter tested in formulation.

## 4. Conclusion

By varying the concentration of Polyox WSR205 and Polyox WSR N12K in the outer barrier layer with gas generating agent the lag time of drug release from the FPRT formulation could be readily modulated. Good correlation was observed between *in vitro* and *in vivo* performance of the compression coated floating-pulsatile release tablet suggesting that the robust and reliable ability to produce a lag time before drug release. This formulation will be useful as a chronopharmaceutical drug delivery system. It can be considered as one of the promising formulation as floating-pulsatile drug release system for bisoprolol.

## Figures and Tables

**Table 1:** Formulations of core tablet.

Ingredients	C1	C2	C3	C4
Bisoprolol fumarate	20	20	20	20
Croscarmellose sodium	6	8	10	12
Magnesium stearate	3	3	3	3
Microcrystalline cellulose	8	8	8	8
Lactose	38	36	34	32

Total (mg)	75	75	75	75

**Table 2:** Preparation of the Floating Pulsatile Release Tablet (FPRT) trial batches individual polymer.

Sr. no.	Ingredients	Formulation codes					
		P1	P2	P3	P4	P5	P6
1	Polyox-205	160	140	120	—	—	—
2	Polyox-N12K	—	—	—	150	130	110
3	NaHCO_3_	45	45	45	45	45	45
4	Citric acid	15	15	15	15	15	15
5	Ca_2_PO_4 _	—	20	40	10	30	50

**Table 3:** 3^2^ full factorial design.

Formulation no.	Coded levels	
	Variable 1	Variable 2
F10	−1	−1
F11	−1	0
F12	−1	+1
F13	0	−1
F14	0	0
F15	0	+1
F16	+1	−1
F17	+1	0
F18	+1	+1

**Table 4:** Concentrations of the variables according to the coded levels used in factorial design.

Variables used	Coded levels		
	−1	0	+1
Polyox WSR205 (in mg)	45	55	65
Polyox WSR N12K (in mg)	65	75	85

**Table 5:** Formulation of batches containing Polyox WSR205 and Polyox N12K as variables according to the 3^2^ full factorial design.

Formulation no.	Polyox WSR205 (mg)	Polyox WSR N12K (mg)	Sodium bicarbonate (mg)	Citric acid (mg)	Core tablet (mg)	Calcium diphosphate (mg)
F10	45	65	50	20	75	40
F11	45	75	50	20	75	30
F12	45	85	50	20	75	20
F13	55	65	50	20	75	30
F14	55	75	50	20	75	20
F15	55	85	50	20	75	10
F16	65	65	50	20	75	20
F17	65	75	50	20	75	10
F18	65	85	50	20	75	—

**Table 6:** Evaluation of floating-pulsatile release tablet (F10–F18).

Formulation no.	Tablet weight (mg)	Tablet thickness (mm)	% drug content	Hardness (Kg/cm^2^)	Swelling index	Buoyancy lag time (sec.)	% drug release
F10	294.12 ± 1.56	2.55 ± 0.07	98.45 ± 0.56	7.5 ± 0.13	188.45 ± 2.34	108 ± 2	99.49 ± 1.32
F11	297.29 ± 2.14	2.64 ± 0.04	97.27 ± 0.75	7.8 ± 0.25	192.66 ± 3.75	109 ± 3	99.23 ± 0.74
F12	298.54 ± 1.35	2.58 ± 0.03	98.17 ± 1.34	7.7 ± 0.06	201.56 ± 2.56	113 ± 5	99.30 ± 0.96
F13	295.23 ± 0.95	2.52 ± 0.05	97.96 ± 0.78	8.1 ± 0.15	206.45 ± 1.34	102 ± 3	99.89 ± 2.01
F14	292.11 ± 1.23	2.44 ± 0.08	98.62 ± 1.56	7.4 ± 0.08	210.55 ± 3.13	116 ± 2	83.60 ± 0.82
F15	294.78 ± 0.87	2.63 ± 0.02	97.39 ± 0.50	7.9 ± 0.12	222.44 ± 1.57	115 ± 2	80.94 ± 0.95
F16	297.89 ± 0.98	2.68 ± 0.10	97.19 ± 2.34	8.2 ± 0.34	226.45 ± 2.18	111 ± 3	75.21 ± 1.74
F17	291.55 ± 1.34	2.57 ± 0.04	98.82 ± 0.34	7.6 ± 0.26	231.61 ± 3.56	110 ± 5	66.96 ± 1.98
F18	298.78 ± 2.15	2.62 ± 0.03	96.98 ± 2.78	7.8 ± 0.13	239.77 ± 2.15	119 ± 5	59.94 ± 2.02
